# Automatic Off-Line Design of Robot Swarms: A Manifesto

**DOI:** 10.3389/frobt.2019.00059

**Published:** 2019-07-19

**Authors:** Mauro Birattari, Antoine Ligot, Darko Bozhinoski, Manuele Brambilla, Gianpiero Francesca, Lorenzo Garattoni, David Garzón Ramos, Ken Hasselmann, Miquel Kegeleirs, Jonas Kuckling, Federico Pagnozzi, Andrea Roli, Muhammad Salman, Thomas Stützle

**Affiliations:** ^1^Université libre de Bruxelles, Brussels, Belgium; ^2^Alma Mater Studiorum, Università di Bologna, Bologna, Italy

**Keywords:** swarm robotics, automatic design, collective behaviors, design methodology, evolutionary robotics

## Abstract

Designing collective behaviors for robot swarms is a difficult endeavor due to their fully distributed, highly redundant, and ever-changing nature. To overcome the challenge, a few approaches have been proposed, which can be classified as manual, semi-automatic, or automatic design. This paper is intended to be the manifesto of the automatic off-line design for robot swarms. We define the off-line design problem and illustrate it via a possible practical realization, highlight the core research questions, raise a number of issues regarding the existing literature that is relevant to the automatic off-line design, and provide guidelines that we deem necessary for a healthy development of the domain and for ensuring its relevance to potential real-world applications.

Although swarm robotics is widely recognized as a promising approach to coordinating large groups of robots (Dorigo et al., 2014; Yang et al., 2018) and has already gained a prominent position in the scientific literature (e.g., see Rubenstein et al., 2014; Werfel et al., 2014; Garattoni and Birattari, 2018; Slavkov et al., 2018; Yu et al., 2018; Li et al., 2019; Xie et al., 2019), a general methodology for designing collective behaviors for robot swarms is still missing (Brambilla et al., 2013). The design problem is particularly challenging because it aims at producing a system that is autonomous, fully distributed, and highly redundant: robots do not have any predefined role and do not rely on any external infrastructure (Beni, 2004; Şahin, 2004). A robot swarm is a loosely coupled system in which the collective behavior of the system results from the local interactions between individuals, and between them and the environment. These interactions cannot be explicitly defined at design time due to the high uncertainty that characterizes the operation of a swarm. As a result, at least in the general case, it is impossible to tell what the individual robots should do so that a desired collective behavior is achieved. This rules out the application of traditional multi-robot systems and software engineering techniques, which rely on formally deriving the individual behaviors of the robots from specifications expressed at the collective level (Brugali, 2007; Di Ruscio et al., 2014; Bozhinoski et al., 2015; Schlegel et al., 2015).

A few methods/tools have been proposed that, under a number of restrictive hypotheses and constraints, support the designer for specific classes of missions (Hamann and Wörn, 2008; Kazadi, 2009; Berman et al., 2011; Beal et al., 2012; Brambilla et al., 2015; Reina et al., 2015; Lopes et al., 2016; Pinciroli and Beltrame, 2016). Also, a few automatic (and semi-automatic) design methods have been proposed that operate under various assumptions (Nolfi and Floreano, 2000; Watson et al., 2002; Duarte et al., 2014; Francesca et al., 2014). For recent discussions, see Francesca and Birattari (2016) and Bredeche et al. (2018).

This paper is intended to be the manifesto of the automatic off-line design of robot swarms. In this approach, the design problem is cast into an optimization problem that is solved off-line—that is, before the swarm is deployed in the target environment. An optimization algorithm searches a space of possible designs with the goal of maximizing an appropriate mission-specific performance measure. Within the design process, the performance of candidate designs explored by the optimization algorithm is assessed via computer-based simulations. Once the optimization algorithm terminates, the selected design is uploaded to the robots and the swarm is deployed in its target environment. In the following, we focus mostly on the development of software but the discussion can be directly extended to the hardware. For example, the automatic off-line design process might optimize the number of robots in the swarm; if the swarm is heterogeneous, select the fraction of robots of type A, B, C,…; fine-tune parameters of hardware or firmware; activate/deactivate or add/remove hardware modules; design chassis, shell, or attachments.

Our vision is that, in a relatively close future, automatic off-line design will be a practically relevant way of realizing robot swarms. Likely, it will not be the only one: other approaches will be available, each with its specific advantages and ideal areas of application, as well as its disadvantages and limitations. Among them, we foresee that a relevant role will be played by manual design, semi-automatic design, automatic on-line design, and hybrid approaches that combine the previous ones. Nonetheless, we expect that the automatic off-line approach will play a major role, both on its own and also as a component of hybrid approaches.

In the automatic off-line approach, robot swarms are generated to perform missions that are sampled from a given class of interest and are sufficiently different from one another to possibly require (or benefit from) a tailored design. An automatic off-line method must operate on the missions of the given class without requiring either mission-specific adjustments, or per-mission human intervention. The notion of a class of missions plays here a central role. It refers to a set of missions, together with a probability measure defined on them, which determines their relative frequency of appearance. Typically, an explicit, closed-form definition of the set of missions and of the probability measure is not available—and is not even needed. Instead, what we have is a stream of missions sampled from the class of interest according to the aforementioned probability measure. The assumption that missions are sampled according to a probability measure gives a formal meaning to the notion of expected performance, as well as to any other statistics one might wish to adopt to describe the aggregate behavior of a design method across the missions of interest. To illustrate the automatic off-line design of robot swarms, it is convenient to sketch one of its possible practical applications.

**Fiorella's swarm gardening**

*(for an artist's rendition, see* *Figure 1**)*

Fiorella owns a robot-swarm gardening business and offers her individually-tailored service to her many customers in the Brussels area. She has a busy schedule: every day, she visits three or four customers with her gardening swarm. Customers book Fiorella's service via a form on her website. Through the form, they ask for one or more specific interventions—e.g., cutting grass, watering flowers. They also provide relevant information on their garden—e.g., size, shape, orientation. The interventions requested and the characteristics of the garden specify the mission that Fiorella's swarm must perform for a specific customer. As the list of possible interventions and characteristics of the garden is huge, the class of possible missions is overwhelmingly large and rather diverse. To provide her customers with the best gardening experience—but also to cut costs and maximize her benefit—Fiorella relies on an automatic off-line method that designs and fine-tunes the behaviors of her swarm specifically for each mission. The design process takes place while Fiorella drives her swarm to the customer's garden: her powerful computers run simulations using the information provided by the customer on the interventions and on the garden. The design process must be performed within a limited amount of time—the time of the ride to the customer's. As Fiorella arrives on the spot, the selected design is uploaded to the robots and the swarm is deployed in the garden. Fiorella cannot intervene in the design process—she drives the van in the meantime. Moreover, due to her tight schedule, Fiorella cannot either test the selected design on the robots before deployment and possibly re-run the design process: once she reaches the customer, robots must be operational. Any per-mission human intervention and any test on the robots in the target environment would be too time consuming and expensive: they would increase costs dramatically and Fiorella would be unavoidably out of business.

**Figure 1:**
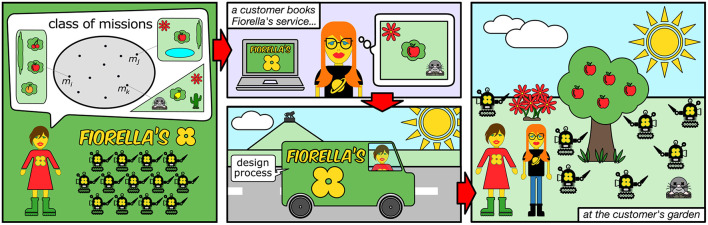
Fiorella's swarm gardening. Fiorella's robots can perform a large class of gardening missions. Through her website, customers book Fiorella's services, specify the interventions to be performed, and provide a description of their garden. On the basis of this information, while Fiorella drives her robots to customers, her algorithms automatically design and fine-tune the behavior of the robots so as to offer a tailored service. When she arrives at a customer location, the gardening swarm is operational and immediately deployed.

Missions in the class of interest can be relatively minor variations of each other—e.g., cut the grass in a small garden; in a large one; in one with a central flower bed. In this case, the behaviors to be produced will be similar, with some minor differences to increase performance or reduce execution time. Missions can also be substantially different in the nature of their goals and require major differences in the behaviors to be realized—e.g., cut the grass; gather dead leaves in a specific place; locate and map mole tunnels.

In Fiorella's example, the central role of the notion of class of missions emerges clearly. Fiorella faces a stream of missions sampled from the possible missions for which customers might demand her intervention—and which her swarm can hopefully accomplish. It is in the repetitive nature of the design problems faced by Fiorella that the significance of automatic design lies. Indeed, if Fiorella had to solve a single design problem (instead of a stream thereof) she could more profitably solve it either manually or via a semi-automatic design method^1^. It is only when one has to solve a stream of design problems that the human intervention might become uneconomical or even unfeasible. Conceiving, implementing, and setting up an automatic design method is in itself an investment of time and resources, which pays off only if the design process is then repeated a sufficient number of times on multiple missions—those of the class for which the automatic design method is conceived. If one had to address a single mission, it would be more sensible to invest time and resources on that specific mission—by adopting an *ad-hoc* manual or semi-automatic approach—rather than on the development of an automatic design method that would be then used only once. For a schematic representation of the automatic off-line design process, see Figure 2.

**Figure 2:**
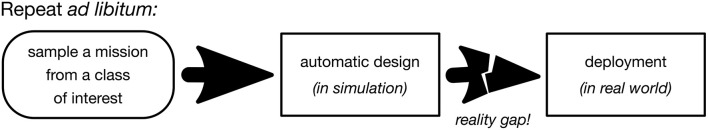
Flowchart diagram of the automatic off-line design process. A mission is sampled from a class of interest. Using computer-based simulations, an automatic design method defines a robot swarm tailored to the mission sampled. Once the automatic design terminates, the swarm is deployed in the target environment and has to cross the so-called reality gap—the possibly subtle but inevitable difference between simulation and reality (Brooks, 1992; Jakobi et al., 1995)—which is among the most challenging issues in automatic off-line design. The process is repeated *ad libitum*. It should be noted that an automatic design method could generate a robot swarm from scratch for every mission sampled or could refine and adapt a solution previously generated for a similar one. It could also, for example, produce a robot swarm by combining and modifying solutions (or partial solutions) contained in a catalog of template solutions that were pre-defined by a human expert. The only condition that needs to be respected for the process to be qualified as automatic off-line design is that such initial (partial) solutions must be selected without any per-mission human intervention and without recourse to tests performed in the target environment.

Fiorella's example allows us to highlight a number of issues and research questions that are relevant to the automatic off-line design of robot swarms.

**Can effective robot swarms be designed automatically via an off-line process?** Can we conceive a method to automatically design a swarm for any mission within a given class? What is the class of missions for which a given method can design an appropriate swarm? How can a given method be generalized to solve a larger class of missions?

**Given a class of missions, which is the most appropriate design method?** What elements or characteristics of a design method influence its ability to handle missions of a given class? Vice versa, what elements/characteristics of a class of missions might suggest that a given method is appropriate to handle them? Which features of a specific mission make it challenging for a given design method? Are these challenging features equally challenging for all possible design methods? Is it possible to match challenging features of missions with characteristics of design methods?

**To what extent a given design method is robust to the so called reality gap—that is, the difference between simulation models and reality?** Is it possible to predict the performance drop that a swarm designed off-line will experience when deployed in the target environment? Are different design methods equally sensitive to the reality gap? What elements/characteristics of a design method make it more or less robust to the reality gap? Can these characteristics be leveraged to engineer a design method that is inherently robust to the reality gap? How should models be devised to be effectively used within an off-line design process?

**How efficient is a design method?** In other terms, how many off-line simulation runs are required to produce an effective design? What elements/characteristics of a design method increase/decrease its efficiency? How well does a given design method behave for a large/small design budget—that is, when allowed to perform few/many off-line simulation runs? Does the efficiency of a design method depend on the specific mission or class of missions considered? What elements/characteristics of a mission determine the minimum size of the design budget needed to produce an effective design? When should a design process be stopped?

This list of questions encompasses many relevant issues but it is by no means exhaustive. For example, other relevant issues would concern the off-line definition of the swarm size (or its spatial density), its impact on performance, and the robustness/scalability of behaviors that are automatically designed.

A body of literature exists that is relevant to the automatic off-line design of robot swarms. The largest share of the design methods described in the relevant literature belong in the neuro-evolutionary domain (Nolfi and Floreano, 2000): robots are controlled by a neural network whose synaptic weights (and possibly the topology) are optimized by an evolutionary algorithm (Quinn et al., 2003; Christensen and Dorigo, 2006; Baldassarre et al., 2007; Trianni, 2008; Hauert et al., 2009; Trianni and Nolfi, 2009; Waibel et al., 2009; Ferrante et al., 2013, 2015; Gomes et al., 2013; Trianni and López-Ibáñez, 2015). For a review of the neuro-evolutionary approach (including also single-robot studies) see Floreano and Keller (2010), Bongard (2013), Bongard and Lipson (2014), Trianni (2014), Doncieux et al. (2015), and Silva et al. (2016). Other approaches depart from neuro-evolution as (i) robots are controlled by software architectures other than neural networks (Hecker et al., 2012; Gauci et al., 2014a; Jones et al., 2016), or (ii) they adopt optimization algorithms other than an evolution algorithm (Pugh et al., 2005), or (iii) both (Francesca et al., 2014, 2015; Gauci et al., 2014b; Kuckling et al., 2018). Besides, a few studies exist that provide insight into the reality gap in the automatic design of robot swarms and/or define methods to handle it (Francesca et al., 2014; Birattari et al., 2016; Ligot and Birattari, 2018). Additionally, a number of methods have been proposed that, although described in single-robot applications, are relevant to the design of robot swarms (Jakobi et al., 1995; Miglino et al., 1995; Floreano and Mondada, 1996; Jakobi, 1997; Bongard and Lipson, 2004; Zagal et al., 2004; Boeing and Braunl, 2012; Koos et al., 2013).

It is our contention that, with only few exceptions, the aforementioned methods have been studied following protocols that were not conceived to directly address the core research questions sketched above. Although these protocols allowed studies which partially addressed those questions, they were conceived to target other questions that are mostly relevant to other domains including, for example, evolutionary biology and the semi-automatic design of robot swarms^1^. In almost the totality of the studies, the focus is on a specific mission that must be performed by a swarm—or, equivalently, on a specific capability that the swarm should acquire and display. The design method is proposed only as a way to achieve the desired collective behavior and is not the protagonist of the study: the study is not structured to highlight its properties and assess its performance. The design method has so little importance that it is not customarily given an identifying name—contrary to what happens in related fields such as machine learning or heuristic optimization. Typically, the design method is tested on a single mission and it is not compared to any alternative. It is rare that a same design method is tested across multiple studies on multiple missions without undergoing any (manually-applied) mission-specific modification. In many studies, the control software produced by a design method is tested only in simulation and no assessment is provided on whether and to what extent it crosses the reality gap satisfactorily. Moreover, design methods survive only the time span of the paper in which they are introduced and their implementation is not routinely made publicly available for further studies, to be possibly performed by a third party. Often, a design method is run iteratively on a single mission. It is run once, the behavior generated is inspected by the designer who then modifies the method itself or the objective function to be optimized—e.g., by adding/removing terms. These activities are then iterated at will until a satisfactory behavior is obtained. In most cases, this iterative process is not detailed in research articles: it is often unclear how many iterations have been performed, what has been measured at each iteration, what modifications have been implemented, what ideas have been tried and then abandoned. In these cases, the research articles present only the final setting that eventually generated the behavior discussed. The iterative process is repeated only once, as it would be difficult to produce independent trials of a process that features a human in the loop. As a result, the robustness and the repeatability of the process are not assessed.

An appropriate protocol to address the aforementioned issues should reflect the following tenets of the research in automatic off-line design: (i) automatic off-line design methods should not be mission-specific and should be able to address a whole class of missions without undergoing any modification; (ii) once a mission is specified, human intervention is not provided for in any phase of the design process. Indeed, research that is intended to be relevant to the automatic off-line design of robot swarms should exclude the case in which design methods are conceived for or are manually adapted to a specific mission—for example, by manually tuning parameters of the optimization algorithm and/or of the control architecture, or by pre-filtering sensor readings on the basis of insight that only a human designer can provide. It should also exclude the case in which, on a per-mission basis, human designers are allowed to inspect (via either simulation or robot experiments) the behavior of an automatically designed swarm and, on the basis of their observations, modify elements of the automatic design process and iterate it at will, until they obtain satisfactory results. In particular, human designers should not be allowed, on a per-mission basis, to use any insight gained through inspection to modify the design method (optimization algorithm, architecture, sensor pre-filtering, etc.); to adapt simulation models; and to amend the objective function by adding/removing terms so as to steer the design process as wished. On the other hand, to effectively contribute to the development of the domain, researchers in the automatic off-line design of robot swarms should pay particular attention to a number of methodological issues. In particular, they should: (a) provide a clear and thorough description of the design methods they propose, including a list of the value of all parameters; (b) precisely characterize the platforms for which these methods can generate control software; (c) clearly identify and name methods for future reference; (d) publish implementations; (e) test methods on multiple missions; (f) identify—at least informally—the class of missions that a method is intended to address; (g) perform comparative studies in which methods under analysis are tested under the same conditions; and (h) run robot experiments to assess robustness to the reality gap. It is our contention that this minimal set of guidelines will allow the domain to grow healthy and thriving so as to eventually prove its practical relevance in real-world applications.

## Author Contributions

All authors contributed to the elaboration of the ideas presented in the paper, read the text, and provided comments. In particular, MBi started the discussion and lead it, he also drafted the paper and coordinated its revision. AL selected/reviewed the relevant literature and contributed to the formulation of the research questions. DB contributed to framing the complexity of the design problem in swarm robotics. The other authors equally contributed to this paper and are listed in alphabetical order.

### Conflict of Interest Statement

The authors declare that the research was conducted in the absence of any commercial or financial relationships that could be construed as a potential conflict of interest.

## Footnotes

^1^By semi-automatic design, we mean an approach in which a human designer is assisted by an optimization process similar to the one of automatic design, but can afford intervening in the process (on a per-mission basis) to guide it according to their insight.

## References

[B1] Baldassarre G. Trianni V. Bonani M. Mondada F. Dorigo M. Nolfi S. (2007). Self-organized coordinated motion in groups of physically connected robots. IEEE Trans. Syst. Man Cybern. B 37, 224–239. 10.1109/TSMCB.2006.881299 17278574

[B2] Beal J. Dulman S. Usbeck K. Viroli M. Correll N. (2012). Organizing the aggregate: languages for spatial computing, in Formal and Practical Aspects of Domain-Specific Languages: Recent Developments, Chap. 16, ed M. Mernik (Hershey, PA: IGI Global), 436–501.

[B3] Beni G. (2004). From swarm intelligence to swarm robotics, in Swarm Robotics, SAB, volume 3342 of LNCS (Berlin: Springer), 1–9.

[B4] Berman S. Kumar V. Nagpal R. (2011). Design of control policies for spatially inhomogeneous robot swarms with application to commercial pollination, in IEEE International Conference on Robotics and Automation – ICRA (Piscataway, NJ: IEEE), 378–385.

[B5] Birattari M. Delhaisse B. Francesca G. Kerdoncuff Y. (2016). Observing the effects of overdesign in the automatic design of control software for robot swarms, in Swarm Intelligence – ANTS, volume 9882 of LNCS (Cham: Springer), 45–57.

[B6] Boeing A. Braunl T. (2012). Leveraging multiple simulators for crossing the reality gap, in Proceedings of the International Conference on Control, Automation, Robotics and Vision – ICARCV (Piscataway NJ: IEEE), 1113–1119.

[B7] Bongard J. Lipson H. (2004). Once more unto the breach: co-evolving a robot and its simulator, in Artificial Life IX: Proceedings of the Conference on the Simulation and Synthesis of Living Systems (Cambridge, MA: MIT Press), 57–62.

[B8] Bongard J. Lipson H. (2014). Evolved machines shed light on robustness and resilience. Proc. IEEE 102, 899–914. 10.1109/JPROC.2014.2312844

[B9] Bongard J. C. (2013). Evolutionary robotics. Commun. ACM 56, 74–83. 10.1145/2492007.2493883

[B10] Bozhinoski D. Di Ruscio D. Malavolta I. Pelliccione P. Tivoli M. (2015). Flyaq: enabling non-expert users to specify and generate missions of autonomous multicopters, in IEEE/ACM International Conference on Automated Software Engineering – ASE (Piscataway, NJ: IEEE), 801–806.

[B11] Brambilla M. Brutschy A. Dorigo M. Birattari M. (2015). Property-driven design for swarm robotics: a design method based on prescriptive modeling and model checking. ACM Trans. Auton. Adapt. Syst. 9, 17.1–28. 10.1145/2700318

[B12] Brambilla M. Ferrante E. Birattari M. Dorigo M. (2013). Swarm robotics: a review from the swarm engineering perspective. Swarm Intell. 7, 1–41. 10.1007/s11721-012-0075-2

[B13] Bredeche N. Haasdijk E. Prieto A. (2018). Embodied evolution in collective robotics: a review. Front. Robot. AI 5:12 10.3389/frobt.2018.00012 PMC7806005 33500899

[B14] Brooks R. (1992). Artificial life and real robots, in Towards a Practice of Autonomous Systems. Proceedings of the First European Conference on Artificial Life (Cambridge, MA: MIT Press), 3–10.

[B15] Brugali D. (2007). Software Engineering for Experimental Robotics, Vol. 30 Berlin; Heidelberg: Springer.

[B16] Christensen A. L. Dorigo M. (2006). Evolving an integrated phototaxis and hole-avoidance behavior for a swarm-bot, in Arfiticial Life – ALIFE (Cambridge, MA: MIT Press), 248–254.

[B17] Di Ruscio D. Malavolta I. Pelliccione P. (2014). A family of domain-specific languages for specifying civilian missions of multi-robot systems, in Proceedings of the 1st International Workshop on Model-Driven Robot Software Engineering – MORSE (York: MORSE workshop), 13–26. Available online at: http://ceur-ws.org/Vol-1319/

[B18] Doncieux S. Bredeche N. Mouret J.-B. Eiben A. E. G. (2015). Evolutionary robotics: what, why, and where to. Front. Robot. AI 2:4 10.3389/frobt.2015.00004

[B19] Dorigo M. Birattari M. Brambilla M. (2014). Swarm robotics. Scholarpedia 9:1463 10.4249/scholarpedia.1463

[B20] Duarte M. Oliveira S. Christensen A. (2014). Evolution of hierarchical controllers for multirobot systems, in Arfiticial Life – ALIFE, eds Sayama H. Rieffel J. Risi S. Doursat R. Lipson H. (Cambridge, MA: MIT Press), 657–664.

[B21] Ferrante E. Duéñez Guzmán E. Turgut A. E. Wenseleers T. (2013). Geswarm: grammatical evolution for the automatic synthesis of collective behaviors in swarm robotics, in Genetic and Evolutionary Computation – GECCO (New York, NY: ACM), 17–24.

[B22] Ferrante E. Turgut A. Duéñez-Guzmán E. Dorigo M. Wenseleers T. (2015). Evolution of self-organized task specialization in robot swarms. PLoS Comput. Biol. 11:e1004273. 10.1371/journal.pcbi.1004273 26247819 PMC4527708

[B23] Floreano D. Keller L. (2010). Evolution of adaptive behaviour in robots by means of darwinian selection. PLoS Biol. 8:e1000292. 10.1371/journal.pbio.1000292 20126252 PMC2811146

[B24] Floreano D. Mondada F. (1996). Evolution of plastic neurocontrollers for situated agents, in From Animals to Animats 4: Proceedings of the Fourth International Conference on Simulation of Adaptive Behavior (SAB) (Cambridge, MA: MIT Press), 402–410.

[B25] Francesca G. Birattari M. (2016). Automatic design of robot swarms: achievements and challenges. Front. Robot. AI 3:29 10.3389/frobt.2016.00029

[B26] Francesca G. Brambilla M. Brutschy A. Garattoni L. Miletitch R. Podevijn G. (2015). AutoMoDe-Chocolate: automatic design of control software for robot swarms. Swarm Intell. 9, 125–152. 10.1007/s11721-015-0107-9

[B27] Francesca G. Brambilla M. Brutschy A. Trianni V. Birattari M. (2014). AutoMoDe: a novel approach to the automatic design of control software for robot swarms. Swarm Intell. 8, 89–112. 10.1007/s11721-014-0092-4

[B28] Garattoni L. Birattari M. (2018). Autonomous task sequencing in a robot swarm. Sci. Robot. 3:eaat0430 10.1126/scirobotics.aat0430 33141726

[B29] Gauci M. Chen J. Li W. Dodd T. J. Groß R. (2014a). Clustering objects with robots that do not compute, in Autonomous Agents and Multiagent Systems – AAMAS (Richland, SC: IFAAMAS), 421–428.

[B30] Gauci M. Chen J. Li W. J Dodd T. Groß R. (2014b). Self-organized aggregation without computation. Int. J. Robot. Res. 33, 1145–1161. 10.1177/0278364914525244

[B31] Gomes J. Urbano P. Christensen A. (2013). Evolution of swarm robotics systems with novelty search. Swarm Intell. 7, 115–144. 10.1007/s11721-013-0081-z

[B32] Hamann H. Wörn H. (2008). A framework of space–time continuous models for algorithm design in swarm robotics. Swarm Intell. 2, 209–239. 10.1007/s11721-008-0015-3

[B33] Hauert S. Zufferey J.-C. Floreano D. (2009). Evolved swarming without positioning information: an application in aerial communication relay. Auton. Robots 26, 21–32. 10.1007/s10514-008-9104-9

[B34] Hecker J. P. Letendre K. Stolleis K. Washington D. Moses M. E. (2012). Formica ex machina: ant swarm foraging from physical to virtual and back again, in Swarm Intelligence – ANTS, volume 7461 of LNCS (Berlin: Springer), 252–259.

[B35] Jakobi N. (1997). Evolutionary robotics and the radical envelope-of-noise hypothesis. Adapt. Behav. 6, 325–368. 10.1177/105971239700600205

[B36] Jakobi N. Husbands P. Harvey I. (1995). Noise and the reality gap: the use of simulation in evolutionary robotics. Lect. Notes Comput. Sci. 929, 704–720. 10.1007/3-540-59496-5_337

[B37] Jones S. Studley M. Hauert S. Winfield A. (2016). Evolving behaviour trees for swarm robotics, in Distributed Autonomous Robotic Systems (Cham: Springer), 487–501.

[B38] Kazadi S. (2009). Model independence in swarm robotics. Int. J. Intell. Comput. Cybern. 2, 672–694. 10.1108/17563780911005836

[B39] Koos S. Mouret J.-B. Doncieux S. (2013). The transferability approach: crossing the reality gap in evolutionary robotics. IEEE Trans. Evol. Comput. 17, 122–145. 10.1109/TEVC.2012.2185849

[B40] Kuckling J. Ligot A. Bozhinoski D. Birattari M. (2018). Behavior trees as a control architecture in the automatic modular design of robot swarms, in Swarm Intelligence – ANTS, volume 11172 of LNCS (Cham: Springer), 30–43.

[B41] Li S. Batra R. Brown D. Chang H.-D. Ranganathan N. Hoberman C.. (2019). Particle robotics based on statistical mechanics of loosely coupled components. Nature 567, 361–365. 10.1038/s41586-019-1022-9 30894722

[B42] Ligot A. Birattari M. (2018). On mimicking the effects of the reality gap with simulation-only experiments, in Swarm Intelligence – ANTS, volume 11172 of LNCS (Cham: Springer), 109–122.

[B43] Lopes Y. K. Trenkwalder S. M. Leal A. B. Dodd T. J. Groß R. (2016). Supervisory control theory applied to swarm robotics. Swarm Intell. 10, 65–97. 10.1007/s11721-016-0119-0

[B44] Miglino O. Lund H. Nolfi S. (1995). Evolving mobile robots in simulated and real environments. Artif. Life 2, 417–434. 10.1162/artl.1995.2.4.417 8942055

[B45] Nolfi S. Floreano D. (2000). Evolutionary Robotics. Cambridge, MA: MIT Press.

[B46] Pinciroli C. Beltrame G. (2016). Buzz: a programming language for robot swarms. IEEE Softw. 33, 97–100. 10.1109/MS.2016.95

[B47] Pugh J. Martinoli A. Zhang Y. (2005). Particle swarm optimization for unsupervised robotic learning, in Swarm Intelligence Symposium – SI (Piscataway, NJ: IEEE), 92–99.

[B48] Quinn M. Smith L. Mayley G. Husbands P. (2003). Evolving controllers for a homogeneous system of physical robots: structured cooperation with minimal sensors. Philos. Trans. R. Soc. A 361, 2321–2343. 10.1098/rsta.2003.1258 14599322

[B49] Reina A. Valentini G. Fernàndez-Oto C. Dorigo M. Trianni V. (2015). A design pattern for decentralised decision making. PLoS ONE 10:e0140950. 10.1371/journal.pone.0140950 26496359 PMC4619747

[B50] Rubenstein M. Cornejo A. Nagpal R. (2014). Programmable self-assembly in a thousand-robot swarm. Science 345, 795–799. 10.1126/science.1254295 25124435

[B51] Şahin E. (2004). Swarm robotics: from sources of inspiration to domains of application, in Swarm Robotics, SAB, volume 3342 of LNCS (Berlin: Springer), 10–20.

[B52] Schlegel C. Lotz A. Lutz M. Stampfer D. Inglés-Romero J. F. Vicente-Chicote C. (2015). Model-driven software systems engineering in robotics: covering the complete life-cycle of a robot. Informat. Technol. 57, 85–98. 10.1515/itit-2014-1069

[B53] Silva F. Duarte M. Correia L. Oliveira S. M. Christensen A. L. (2016). Open issues in evolutionary robotics. Evol. Comput. 24, 205–236. 10.1162/EVCO_a_00172 26581015

[B54] Slavkov I. Carrillo-Zapata D. Carranza N. Diego X. Jansson F. Kaandorp J. (2018). Morphogenesis in robot swarms. Sci. Robot. 3:eaau9178 10.1126/scirobotics.aau9178 33141694

[B55] Trianni V. (2008). Evolutionary Swarm Robotics. Berlin: Springer.

[B56] Trianni V. (2014). Evolutionary robotics: model or design? Front. Robot. AI 1:13 10.3389/frobt.2014.00013

[B57] Trianni V. López-Ibáñez M. (2015). Advantages of task-specific multi-objective optimisation in evolutionary robotics. PLoS ONE 10:e0136406 10.1371/journal.pone.0136406 26295151 PMC4546428

[B58] Trianni V. Nolfi S. (2009). Self-organizing sync in a robotic swarm: a dynamical system view. IEEE Trans. Evol. Comput. 13, 722–741. 10.1109/TEVC.2009.2015577

[B59] Waibel M. Keller L. Floreano D. (2009). Genetic team composition and level of selection in the evolution of multi-agent systems. IEEE Trans. Evol. Comput. 13, 648–660. 10.1109/TEVC.2008.2011741

[B60] Watson R. Ficici G. S. Pollack J. (2002). Embodied evolution: distributing an evolutionary algorithm in a population of robots. Robot. Auton. Syst. 39, 1–18. 10.1016/S0921-8890(02)00170-7

[B61] Werfel J. Petersen K. Nagpal R. (2014). Designing collective behavior in a termite-inspired robot construction team. Science 343, 754–758. 10.1126/science.1245842 24531967

[B62] Xie H. Sun M. Fan X. Lin Z. Chen W. Wang L. (2019). Reconfigurable magnetic microrobot swarm: multimode transformation, locomotion, and manipulation. Sci. Robot. 4:eaav8006 10.1126/scirobotics.aav8006 33137748

[B63] Yang G.-Z. Bellingham J. Dupont E. P. Fischer P. Floridi L. Full R. (2018). The grand challenges of Science Robotics. Sci. Robot. 3:eaar7650 10.1126/scirobotics.aar7650 33141701

[B64] Yu J. Wang B. Du X. Wang Q. Zhang L. (2018). Ultra-extensible ribbon-like magnetic microswarm. Nat. Commun. 9:3260. 10.1038/s41467-018-05749-6 30131487 PMC6104072

[B65] Zagal J. C. Ruiz-Del-Solar J. Vallejos P. (2004). Back to reality: crossing the reality gap in evolutionary robotics. IFAC Proc. Vol. 37, 834–839. 10.1016/S1474-6670(17)32084-0

